# Discovering paracrine regulators of cell type composition from spatial transcriptomics using SPER

**DOI:** 10.1093/bioadv/vbag011

**Published:** 2026-01-19

**Authors:** Tianxiao Zhao, Adam L Haber

**Affiliations:** Institute of Systems Genetics, New York University Grossman School of Medicine, New York, NY, 10016, United States; Department of Environmental Health, Harvard T.H. Chan School of Public Health, Boston, MA, 02115, United States

## Abstract

**Motivation:**

A defining characteristic of biological tissue is its cell type composition. Many pathologies and chronic diseases are associated with perturbations from the homeostatic composition, and these transformations can lead to aberrant or deleterious tissue function. Spatial transcriptomics enables the concurrent measurement of gene expression and cell type composition, providing an opportunity to identify transcripts that co-vary with and potentially influence nearby cell composition. However, no method yet exists to systematically identify such intercellular regulatory factors.

**Results:**

Here, we develop Spatial Paired Expression Ratio (SPER), a computational approach to evaluate the spatial dependence between transcript abundance and cell type proportions in spatial transcriptomics. We demonstrate the ability of SPER to accurately detect paracrine drivers of cellular abundance using simulated data. Using publicly available spatial transcriptomics data from mouse brain and human lung, we show that genes identified by SPER show statistical enrichment for both extracellular secretion and participation in known receptor-ligand interactions, supporting their potential role as compositional regulators. Taken together, SPER represents a general approach to discover paracrine drivers of cellular compositional changes from spatial transcriptomics.

**Availability and implementation:**

The methods are implemented in R and available at: https://github.com/TianxiaoNYU/SPER.

## 1 Introduction

Given the specialized character of each cell type, the distribution of types that compose each biological tissue is fundamental to mediating physiological functions. Accordingly, compositional changes, such as influx of immune cells or metaplasia of fibroblasts, are tightly regulated by paracrine signaling networks, and these networks are fundamental organizing principles of biological tissues (Meizlish *et al.* 2021). Many diseases involve this type of change in corresponding tissues: in Alzheimer’s disease for example, neuropathology is associated with aberrant proliferation of microglia and astrocytes (Johnson *et al.* 2021), which may be mediated by paracrine CX3CL1/CX3CR1 signaling (Pawelec *et al.* 2020); in asthma, airway remodeling driven by IL-13 leads to increased numbers of smooth muscle and goblet cells accompanied by the abnormal recruitment of immune cells (Knoop and Newberry 2018). Triggering factors, like environmental exposures (Carr *et al.* 2016) and microenvironmental signals (Ansell and Vonderheide 2013), can induce cellular composition changes. To gain insight into the normal physiology of tissue composition and transformations associated with disease, it is essential to understand which signals regulate the compositions of cell types in a tissue.

Spatial transcriptomics (ST) reveals (i) paracrine ligand-receptor expression, (ii) cell type composition patterns, and (iii) spatial relationships among transcripts and cell populations. Widely used, cost-effective methods, including ST Visium (10X Genomics 2019) and Slide-seq (Rodriques *et al.* 2019), capture spatial information in voxels containing multiple cells, making the absolute number of cells in each voxel uncertain. To address this, recovering the relative proportions of component cell types, can be straightforwardly achieved by available deconvolution algorithms. Probabilistic-based approaches, like RCTD (Cable *et al.* 2022), cell2location (Kleshchevnikov *et al.* 2022), and SONAR (Liu *et al.* 2023), model the expression data with certain distributions under a Bayesian likelihood framework. All these methods produce data which has the constraint that in each spot all proportions sum to 1, and thus their output is compositional. When combined with gene expression data, this type of spatial compositional data can potentially be used to discover paracrine drivers of tissue transformation, but no computational tools yet exist to do so.

In lieu of appropriate methods, several studies have used classical statistical correlations, such as Pearson’s R and Spearman’s ρ, to identify such signals in spatial transcriptomics data (Park *et al.* 2021, Tuong *et al.* 2021, Grisanti Canozo *et al.* 2022). However, spatial compositional data possesses unique characteristics that requires tailored analytical approaches (Baker *et al.* 2023), as treating each individual cell type’s composition as an independent measurement can result in spurious correlations (Tolosana-Delgado *et al.* 2019). Additionally, classical metrics face other limitations in this context. For cell type A, transcriptional signals with spatial dependence on its composition fall into two categories. The first includes A-type “marker” genes, which are specifically expressed by A and exhibit a spatial relationship with its composition simply because A cells are their primary source. The second, and more intriguing, category includes genes not expressed by A cells but whose spatial patterns correlate with A’s composition, indicating that they are instead produced by neighboring, non-A cells. These genes are potential paracrine regulators influencing A’s distribution and abundance.

Machine learning methods have also been applied to spatial transcriptomics to model spatial patterns and cell-cell interactions. For example, MISTy (Tanevski *et al.* 2022) builds multiple components to model the spatial contexts and extracts the spatial relationships between spatial marker genes. SpatialDM (Li *et al.* 2023a) uses a bivariate Moran’s statistic to identify the direct spatial association between ligand-receptor pairs, while SpaTalk (Shao *et al.* 2022) analyzes cell-level ligand-receptor interactions by testing for enrichment in co-expressed spots. Although these methods are powerful approaches to analyze the spatial distributions of transcriptional signals, most of them, as well as correlation metrics and methods like scHOT (Ghazanfar *et al.* 2020) and SpatialCorr—primarily focus on direct spatial associations between gene pairs or neighboring cells. Consequently, they are not designed to capture indirect or compositionally mediated spatial dependencies, such as diffusible paracrine factors whose influence on cell type abundance may extend beyond immediate contact zones. Moreover, none of these approaches explicitly model cell-type composition or provide a mechanism to detect regulators that drive compositional changes across tissue space. SPER fills this methodological gap by directly quantifying how the spatial expression of genes predicts the local distribution of cell types, thereby identifying putative paracrine regulators that may not correspond to known ligand–receptor pairs.

To address this challenge, we introduce Spatial Paired Expression Ratio (SPER), a computational framework for detecting paracrine signals regulating target cell type composition, even when these signals exhibit non-overlapping spatial dependence. SPER is inspired by the pair correlation function (radial distribution) from physics, which quantifies spatial correlations by estimating the likelihood of one particle appearing at a given distance from another (Satoh 2003). To denoise the spatial compositional signal while preserving its inherent structure, we apply co-kriging, a geostatistical method (Myers 1982), prior to computing pair correlations to identify the transcripts most strongly co-varying with spatial compositions.

In following sections, we describe the SPER algorithm and demonstrate, using simulated data, that it substantially outperforms all other applicable metrics: Pearson correlation, Spearman correlation, and Earth Mover’s Distance (EMD). Applying SPER to published ST profiles from adult mouse brain and human lung, we find that SPER-identified genes are significantly enriched for transcripts encoding extracellularly secreted proteins and those involved in receptor-ligand interactions, supporting their potential roles in paracrine signaling. SPER also identified novel, biologically significant interactions in the mouse brain data, such as identifying the neuropeptide precerebellin (Cbln1) as a novel factor associated with abundance of GABA-ergic Meis2+ neuron expressing its cognate receptor Nrxn2, and the Wnt ligand R-spondin 3 (Rspo3) linked to the same neurons expressing its receptor Lgr5. In human lung data, SPER detects the fibroblast-derived signal fibrillin-1 (FBN1) associated with alveolar type II (AT2) cells, which express the integrin αvβ6 (ITGB6), one of the known receptors for FBN1. Notably, these putative paracrine interactions were not identified by existing computational methods. Taken together, our findings establish SPER as a general and effective approach for identifying putative regulators of cell type composition from spatial transcriptomics data.

## 2 Methods

### 2.1 SPER: spatial paired expression ratio

In general, SPER algorithm requires normalized spatial transcriptomics data $E_{n\times m}$ (gene expression levels normalized by their global average across all spots), a spot distance matrix $M_{n\times n}$, and cell-type compositional data $C_{k\times n}$ as input, where n is the number of spots, m as the number of genes and k as the number of cell types. As the first step, the distance matrix of spots M is partitioned into l adjacency matrices $A_{i}$, indicating whether two spots lie within a specific distance range ${d_{i-1}\;∼d}_{i}$ for i ∈ {0, 1, …, l} with $d_{0}=0$. Consequently, M can be estimated as the summarization of ${d_{i}A}_{i}$, effectively binning distances into discrete groups. By default, distance bins correspond to the spacing between two adjacent spots, with distances exceeding a predefined limit capped at that value. Each adjacency matrices $A_{i}\in {\text{Boolean}}^{n\times n}$ is normalized by multiplying a diagonal matrix $F_{i,\;n\times n}$, where each element is the reciprocal of each row’s sum of $A_{i}$. Then, they are multiplied with $E_{n\times m}$ and $C_{k\times n}$ as:


$$F_{i}=\text{diag}\;(\{\frac{1}{\sum_{k=1}^{n}A_{i,\;j,k}}{\}}_{j=1}^{n}),\;R_{i}=C\times A_{i}\times F_{i}\times E$$

where $R_{i}$ represents the k×m paired expression ratio matrix at the distance range ${D_{i-1}\;∼\;D}_{i}$. Finally, a weight function $\Phi =(\varphi_{1},\;\ldots ,\;\varphi_{l})$ is applied on each gene/cell type pair across $R_{1},\;\ldots ,\;R_{l}$. The default weight function follows a Poisson distribution $\text{Pois}(\overset{-}{\varphi })$ which has an extra penalty on overlapping and distal regions, where the parameter $\overset{-}{\varphi }$ is chosen based on the potential functional distance of paracrine signal. Eventually, we can get the SPER score matrix S as:


$$S=\sum_{i=1}^{l}{\varphi_{i}R}_{i}=\sum_{i=1}^{l}\varphi_{i}CA_{i}F_{i}E$$

### 2.2 Permutation-based significance testing

For each gene–cell-type pair, SPER computes an empirical null distribution of SPER scores by randomly permuting gene expression values across spatial coordinates while maintaining the underlying spatial configuration of cell-type compositions. The empirical *P* value is calculated as the proportion of permuted SPER scores greater than or equal to the observed score. By preserving spatial autocorrelation during permutation, this approach ensures that significance estimates reflect realistic spatial dependencies rather than random noise.

### 2.3 Spatial ST data simulation

The simulations are created by the following steps: (i) A 80 × 80 grids is created and its distance matrix is calculated; (ii) Cell type labels are assigned to each tile of the grid, which follows different mechanisms in three scenarios; (iii) For each cell type, we create the corresponding marker genes and simulated their expression levels across all cells. Corresponding cell types will have higher counts of their markers with their counts following Poisson distributions Pois(λ) where λ∼Unif(15, 30). Other genes follow Poisson distributions Pois(λ′) where λ′∼Unif(0, 3). (iv) The compositional data is computed from marker genes’ expression level. The count of each cell type within a grid is sampled from another Poisson distribution. The final cell-type proportions are the fractions of each cell types within the tiles. Mathematically it can be shown as below, where ${\tilde{y}}_{g,k}$ is the expression level of gene g at tile k, ${\tilde{c}}_{i,k}\;$is the proportion of cell type i at tile k, α is the scale factor:


$${\tilde{y}}_{g,k}∼\mathrm{Pois}(\lambda_{g,k}),\;c_{i,k}∼\text{Pois}(\sum_{g\;\in \;\text{marker}\;\text{of}\;i}\alpha {\lambda_{g,k}\tilde{y}}_{g,k}),\;{\tilde{c}}_{i,k}=\frac{c_{i,k}}{\sum_{i}c_{i,k}}$$

To simulate realistic spatial dependencies, we applied Markov random field algorithm from the R package “mrf2d” ensuring spatial dependence between source and target cells, with target cells exhibiting greater spatial autocorrelation. The single-source scenario simulated one pair of source and target cells and two control cell types, while the multi-source having two sources, one target, and two control cell types. Pearson and Spearman correlations, along with SPER scores, were calculated across three simulation conditions. The corresponding R codes for simulation can be found in the provided online materials.

### 2.4 Collection and pre-processing of scRNA-seq reference and Visium data

For the mouse brain data, the scRNA-seq data were collected and pre-processed by Tasic, B., et al. in the Allen Institute for Brain Science (Tasic *et al.* 2016). The raw data was normalized by Seurat using SCTransform (Stuart *et al.* 2019). Cell types with count less than 10 was removed from the data. For each cell type, their marker genes were then identified by Seurat using MAST one-versus-all test, with fold change >2, adjusted *P* value <10–30, fraction of expressing cells >70%, while other parameters remained at default settings (Finak *et al.* 2015). Mouse brain spatial transcriptomics data were obtained from the 10X Genomics Visium public database (Genomics 2019). The raw count matrix was normalized using Seurat’s SCTransform function with default settings. Physical distances between cells were computed from provided spatial coordinates and the scale factor in the metadata. Processed human lung scRNA-seq and deconvolved Visium data were collected from the Lung Cell Atlas. The human breast cancer datasets were collected from Wu *et al.* (2021), which include both 10X Visium spatial transcriptomics data and matched single-cell RNA-seq references. Both datasets were normalized using Seurat, and cell-type deconvolution was performed using RCTD to estimate spot-level cell-type compositions.

### 2.5 Cell-type deconvolution and co-kriging modeling

Cell-type spatial composition was estimated using RCTD full mode (Cable *et al.* 2022) with the mouse reference data. We selected the 10 most prevalent cell types across all spots: L2/3 IT, L5 IT, Meis2, L4, L6 IT, Oligo, Pvalb, L6 CT, Sst, and L6b, while all other cell types were grouped as “Other.” The compositional matrix was further denoised using co-kriging. Log-ratio variograms were computed using 15 distance bins of 120 μm each. To model inter-cell-type variograms, we applied a compositional linear model of spatial coregionalization incorporating a nugget effect term and three Gaussian variogram terms with effective ranges of 600, 1300, and 3000 μm, representing short-, mid-, and long-range interactions, respectively.

### 2.6 SPER calculation

In the Visium mouse brain and human lung example, we used the normalized ST data and cell-type spatial compositional data as the SPER inputs. Calculation was performed using SPER R package. The weight function is derived from a Poisson distribution (λ = 2.5) to give penalty on overlapping and distal regions while keeping its sum as 1, where the Poisson parameter 2.5 represents 180 nm in reality as the assumed average functional distance of paracrine signals. The choice of weight function is not unique, as users can manually fine-tune it to match the desired functional distance of the signals. The final SPER score ${\tilde{s}}_{i,j}$ was calculated as:


$${\tilde{s}}_{i,j}=\frac{s_{i,j}}{\exp(p_{i,j}-1)}$$

where $s_{i,j}$ stands for the raw SPER score and $p_{i,j}$ for the expression prevalence of gene i and cell type j.

### 2.7 Collection of extracellular gene set and ligand-receptor pair data

To identify the subcellular locations, we used the all channels integrated version of mouse extracellular gene set that includes knowledge, experiments, text mining, and predictions channel from the COMPARTMENTS database (Binder *et al.* 2014). Genes whose locations are in the extracellular region and its confident level greater than 3 are kept. The ligand-receptor interaction dataset is collected from FANTOM5 ligand-receptor datasets (Ramilowski *et al.* 2015) and CellPhoneDB database (Efremova *et al.* 2020).

### 2.8 Calculating correlations and EMD of gene-cell type pairs

The Pearson and Spearman correlations are calculated between the spatial compositional data after co-kriging modeled and the spatial transcriptomics data. The same matrices, but size reduced, are used to calculate the EMD in comparing the computing complexity. We use the physical distance between spots as the ground distances, and the proportions of each cell-type composition are inputted as the weights when comparing to a gene’s spatial distribution. R package “emdist” is used to calculate EMD in the simulations.

### 2.9 Spatial-dependent signal detection

The SPER score matrix, Pearson correlation matrix, and Spearman correlation matrix are used to filter the candidate genes and spatial-dependent signals with the help of scRNA-seq data as the reference of expression levels. When studying the enrichment of extracellular and ligand genes, we used the hypergeometric test to see if they are enriched within the gene set given thresholds. In both cases, the population size is 13 436; the number of successes in the population is 1784 for the extracellular and 1888 for ligands. The sample size and the number of successes in the sample are dependent on the expression-level and measurement-level thresholds. If the *P* values calculated from the tests are less than .05, we will take it as significantly enriched.

### 2.10 Ablation study of co-kriging

To assess the contribution of co-kriging in modeling cell type composition and SPER’s sensitivity, we conducted an ablation sensitivity analysis. Following the same procedure, we applied SPER to raw compositional estimates from RCTD, with and without co-kriging to stabilize and model the data. We benchmarked performance using statistical enrichment of extracellular localization and paracrine ligand participation (Fig. 4, available as supplementary data at *Bioinformatics Advances* online). As expected, co-kriging reduced noise in compositional estimates, leading to increased detection of relevant transcripts. Specifically, 9 out of 10 cell types exhibited more transcripts with extracellular localization, and 8 out of 10 showed an increase in putative paracrine ligands.

## 3 Results

### 3.1 SPER scores paracrine signals by evaluating spatial dependence between genes and cell types

Spatial Paired Expression Ratio (SPER) is a computational framework designed to uncover paracrine signals that regulate cell type compositions within ST datasets. Unlike classical correlation-based approaches that capture trivial spatial associations, SPER explicitly models the spatial interplay between genes—potentially encoding paracrine signal molecules—and cell types within a biologically relevant range (∼250–300 μm or 25 cell diameters) (Francis and Palsson 1997). To detect these interactions, SPER employs the pair correlation function evaluating how a gene’s expression dependence on a target cell type varies with distance to identify strong paracrine spatial signals.

**Figure 1:**
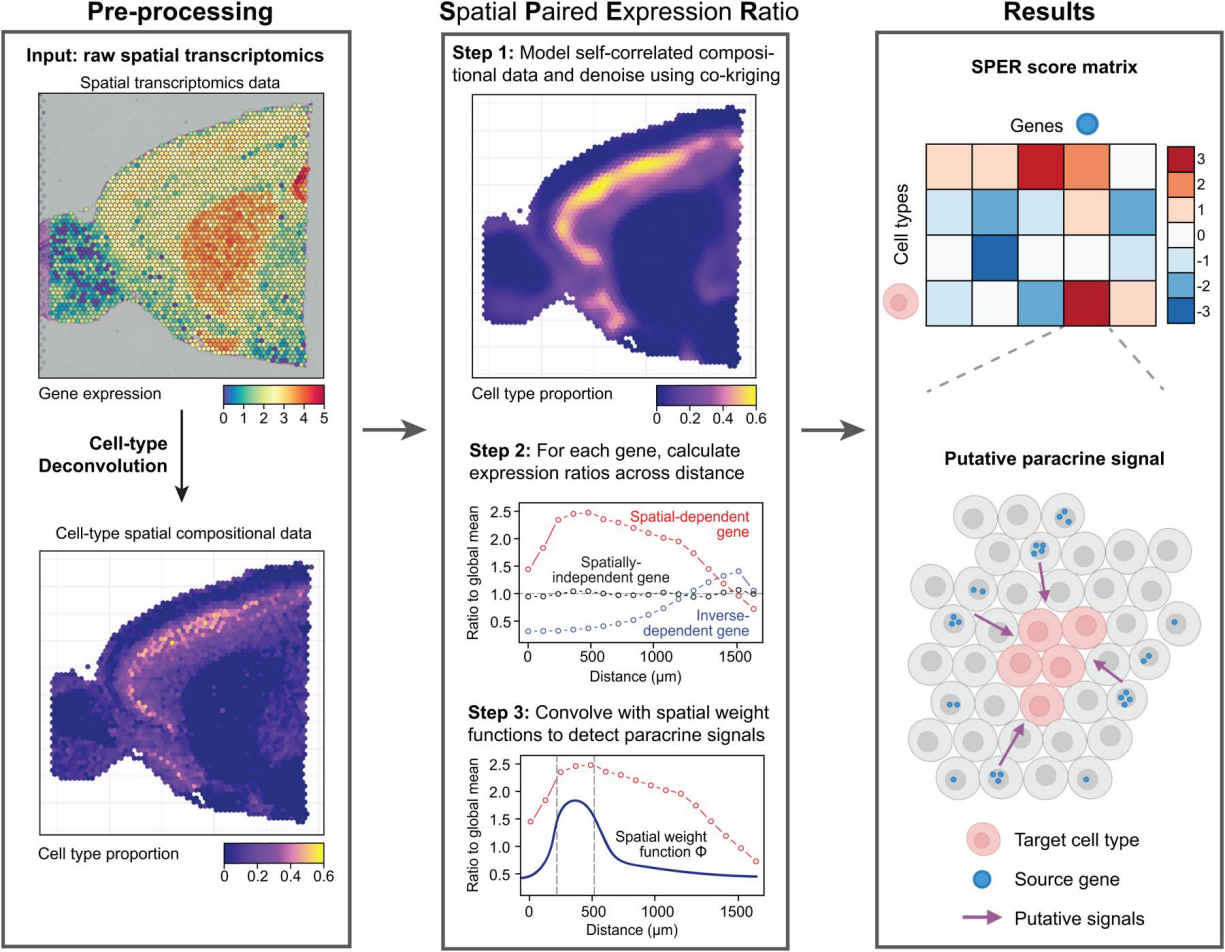
Schematic of spatial paired expression ratio (SPER) algorithm. The workflow of SPER: (Left) Pre-processing: SPER requires three different inputs: spatial transcriptomics data, spatial cell-type compositional data, and the spot-spot distance matrix. Any cell-type deconvolution method can be used to generate the spatial compositional data. (Center) Core algorithms: SPER models the spatial dependence between genes and their potential target cell types by measuring the expression changes over the interaction distance of a cell type-gene pair. As the first step, we apply co-kriging algorithms to model and de-noise the spatial compositional data. Next, we evaluate the spatial association by calculating a gene’s ratios to its mean expression over the distance toward the target cell type. These ratios are defined as the paired expression ratios, and a spatial weight function on these ratios is applied at the last step to emphasize paracrine signal patterns. (Right) Results: the algorithm generates a SPER score matrix as the final output, where higher scores indicate a stronger confidence of this cell type-gene pair as a putative paracrine signal, with a schematic example transcript cell-type pair shown (bottom right)

SPER integrates three key components: spatial gene expression, spatially resolved cell-type compositions (from deconvolution), and spot-to-spot spatial distances (Fig. 1). It first applies geostatistical techniques, specifically co-kriging, to denoise compositional data while preserving spatial dependencies. For each gene-cell type pair, SPER calculates a paired expression ratio—the gene’s expression relative to its global average—across distance lags. This ratio forms a spatial density distribution, which is convolved with a spatial weight to prioritize biologically relevant interaction scales. After accounting for gene expression prevalence in the target cell type, SPER generates a score matrix that distinguishes between direct spatial associations and non-overlapping dependencies indicative of paracrine regulation.

While no existing methods specifically mine ST data for paracrine signals affecting cell composition, geostatistics provides a foundation for analyzing spatially compositional data. Originally developed for mining applications(Krige 1994), co-kriging is an interpolation approach that models’ compositionality as well as spatial dependance between components of a mixture. It uses kernel functions to describe the pattern of variograms between each pair of components as a function of geographic lag distance (Tolosana-Delgado *et al.* 2019). SPER utilizes co-kriging to de-noise spatial compositional data, model variograms between cell type proportions, and adjust for spatial autocorrelation. It then computes paired expression ratios across all gene-cell type pairs using spatial distance information from ST spot coordinates. Finally, SPER applies a weight function to integrate these ratios over relevant length scales, producing a scalar score that quantifies the spatial relationship between each gene and cell type (Fig. 1 and Section 2). To evaluate the statistical significance of observed SPER scores, we implemented a permutation-based framework that generates empirical null distributions for each gene–cell-type pair while preserving spatial structure. This approach allows for rigorous significance assessment and false discovery rate control when identifying candidate paracrine regulators.

### 3.2 SPER detects simulated paracrine drivers of cell type composition

**Figure 2:**
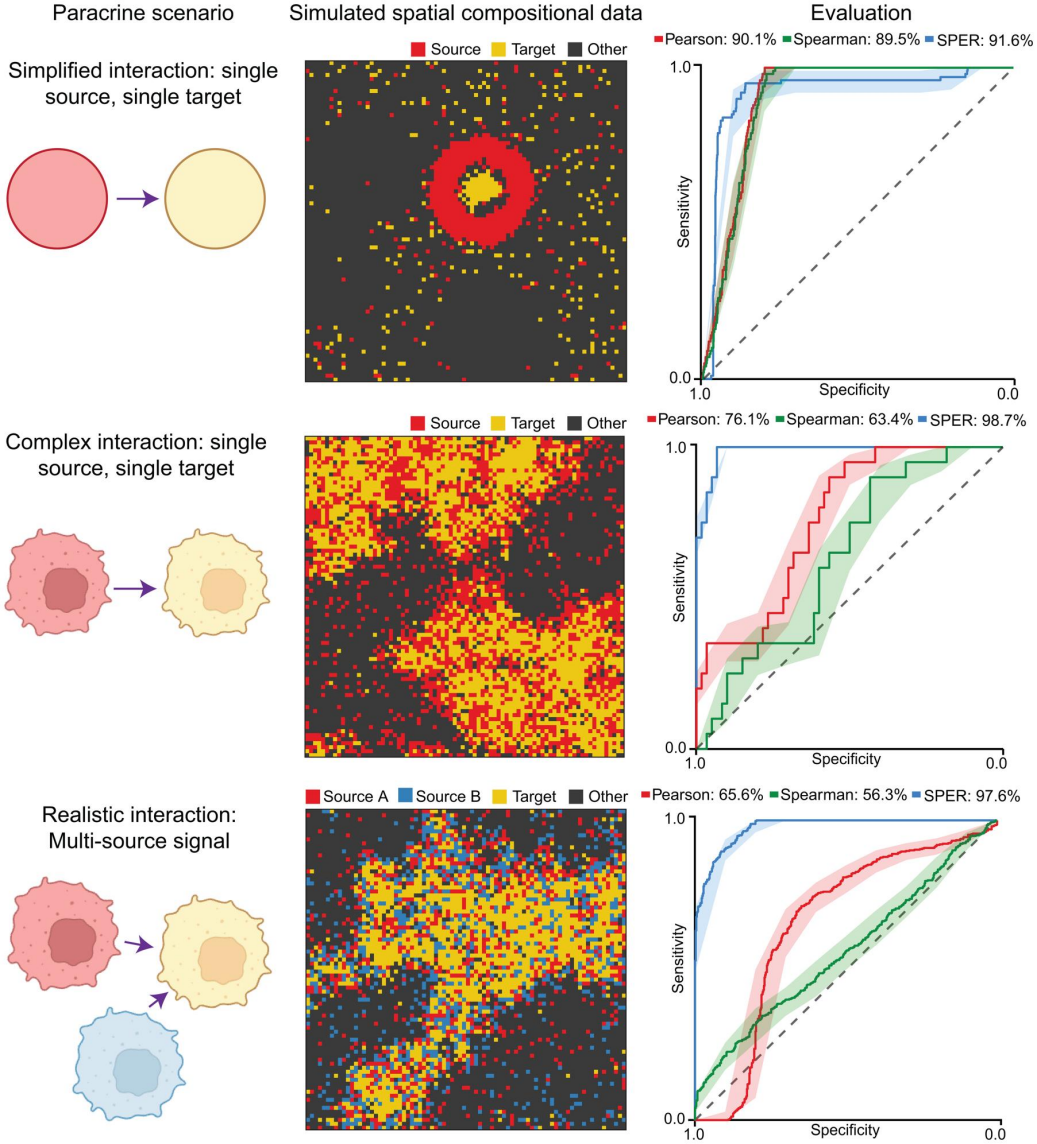
Evaluation of SPER on different simulated spatial transcriptomics data. Left column: Schematic of the two paracrine signal patterns—single-source and multi-source—simulated in this study. Middle column: Visualizations of representative simulated spatial compositional data for each pattern type (rows) in an 80 × 80 grid, with each tile colored by its major cell type (color legend). In the realistic scenarios (bottom two rows), Markov random fields were used to simulate the spatial dependence between the sources and target. Right column: Area under ROC curve (AUROC) values are calculated to evaluate how well each metric distinguishes simulated paracrine signals from other spatially unrelated genes.

To assess SPER’s ability to detect paracrine regulators of cell composition, we applied it to three simulated ST datasets (Fig. 2, Section 2) and compared it to traditional spatial co-variation methods: Pearson correlation, Spearman correlation, and Earth Mover’s Distance (EMD) (Rubner *et al.* 1998). Among simulated expression patterns, cell type-specific marker genes were expressed only in corresponding cells, while paracrine signal genes followed spatial distributions similar to but distinct from their target cell types, expressed by a single source cell type or jointly by multiple types. To model paracrine signaling, we created two scenarios: the single-source, and multi-source patterns (Fig. 2, left), using Markov Random Fields for realistic simulations (Section 2). We applied four different metrics—SPER, Pearson and Spearman correlation, and Earth Mover’s Distance (EMD). While most completed in minutes, EMD’s high computational cost made it impractical for real data, leading to its exclusion.

To enhance biological realism, we developed a Markov Random Field-based simulation where target cell abundance remains spatially associated with, yet distinct from, ligand distribution (Freguglia and Garcia 2022) (Fig. 2, top row). This spatial dependency model captures local heterogeneity, partial overlaps between source and target cells, and stochastic neighborhood variation that reflect realistic tissue organization. We further extended this approach to a multi-source scenario, where target cells are surrounded by two distinct source cell types (Fig. 2, bottom row), simulating complex paracrine contexts such as cooperative or competing signals from multiple niches. In both scenarios, gene expression levels were generated using a hierarchical generative model that introduced random variability across genes and spatial locations, ensuring non-deterministic spatial patterns.

To evaluate method specificity, we also simulated uniformly distributed genes unrelated to any cell type, including the target cell markers. This design allows benchmarking of methods based on their ability to distinguish true paracrine signals from spatially unstructured controls. We repeated each simulation 10× to generate robust results. Given the output scores from each method, we calculated the ROC curve of discriminating paracrine signal genes from other genes. Among all methods, SPER consistently showed the best performance, accurately detecting signals in all three scenarios with AUROC scores from 0.92 to 0.99. Pearson and Spearman correlations, which did well in the simplified scenario, failed to capture paracrine signals in realistic simulations with AUROC scores from 0.56 to 0.76. Taken together, our simulation studies demonstrate the ability of SPER to detect paracrine signals that might be missed by other traditional metrics, especially in a more complex and realistic settings.

### 3.3 SPER outperforms classical metrics in detecting potential paracrine signals in real ST data

**Figure 3:**
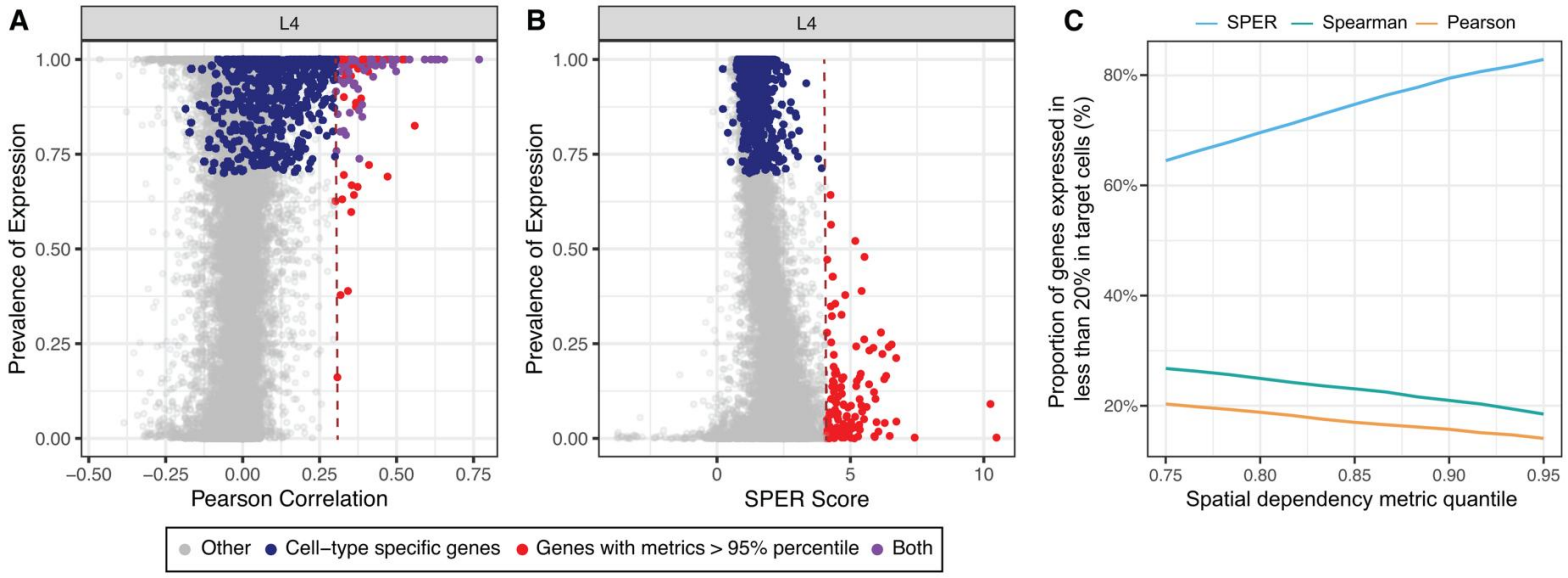
SPER detects non-trivial spatial relationships. (A and B) Scatter plots show the distribution of Pearson correlations (A) and SPER scores (B) with the proportion of layer 4 (L4) cortical neurons in which each gene (dot) is detected in scRNA-seq profiles. The dark blue dots mark the marker genes for each cell type. (C) Proportion of top-ranked (above the given percentile, *x* axis) genes that are not trivially expressed by the target cell type (defined as expression prevalence less than 20% in the target cell type) for each metric (color legend, top).

To evaluate its performance on real data we next applied SPER and the other metrics to ST profiles of mouse brain generated using 10X Visium (Genomics 2019). After preprocessing, 18 270 unique genes were detected across 2695 spots sampled from a sagittal anterior mouse brain section. Based on benchmarking studies evaluating performance on this task (Li *et al.* 2023, Sang-Aram *et al.* 2024), we selected RCTD (Cable *et al.* 2022), which consistently ranks among the top-performing methods, in this study to estimate the cell type composition with a matched scRNA-seq as the reference (Tasic *et al.* 2016) (Fig. 1, available as supplementary data at *Bioinformatics Advances* online). SPER’s co-kriging models showed good fits to the observed variograms of top ten cell types, and visualizing the compositional data after co-kriging demonstrated its ability to remove noise (Fig. 2, available as supplementary data at *Bioinformatics Advances* online). With the reference data, we defined the cell-type-specific marker genes for each of these 10 cell types in Section 2. Given the cell type source of these transcripts, we expect their expression distribution in the ST data to be spatially strongly coupled to the abundance of that cell type. Using Pearson and Spearman correlations, we confirmed that these marker genes showed significant spatial dependency to their corresponding cell types (Fig. 3, available as supplementary data at *Bioinformatics Advances* online).

Our goal is to identify paracrine signals—genes with strong spatial association to a target cell type but low expression within it, suggesting expression by neighboring source cells. To enhance detection, SPER incorporates the gene expression prevalence matrix from scRNA-seq data. To visualize the extent to which putative paracrine transcripts were enriched in each metrics top ranked genes, we plotted the metric scores against the proportion of cells expressing each gene in the reference scRNA-seq data (Tasic *et al.* 2016) for each cell type-gene pair (Fig. 3; Fig. 5, available as supplementary data at *Bioinformatics Advances* online). In L4 (Layer 4) neurons for example (Fig. 3A and B), all genes above the 95th percentile of Pearson correlation also showed high expression prevalence in L4 neurons; and so, it detected just three putative paracrine signals whose expression prevalence less than 0.5 at the 95th percentile threshold, indicating that virtually all transcripts associated with L4 neurons are expressed by them, and many of them are trivially L4 marker genes. On the other hand, in the case of SPER, there are no marker genes within *P* value threshold of .05, and the majority meet our baseline criterion to be putative paracrine transcripts, which we define as genes whose expression prevalence is less than 20% in the target cell type. This trend was consistent across all cell types after varying the metric percentile thresholds from 75th to 95th, where SPER detected markedly more putative paracrine genes meeting this criterion (Fig. 3C), supporting SPER’s capability to detect putative paracrine regulators of cell type composition.

### 3.4 Transcripts with high SPER scores are enriched for extracellular proteins and paracrine ligands

**Figure 4:**
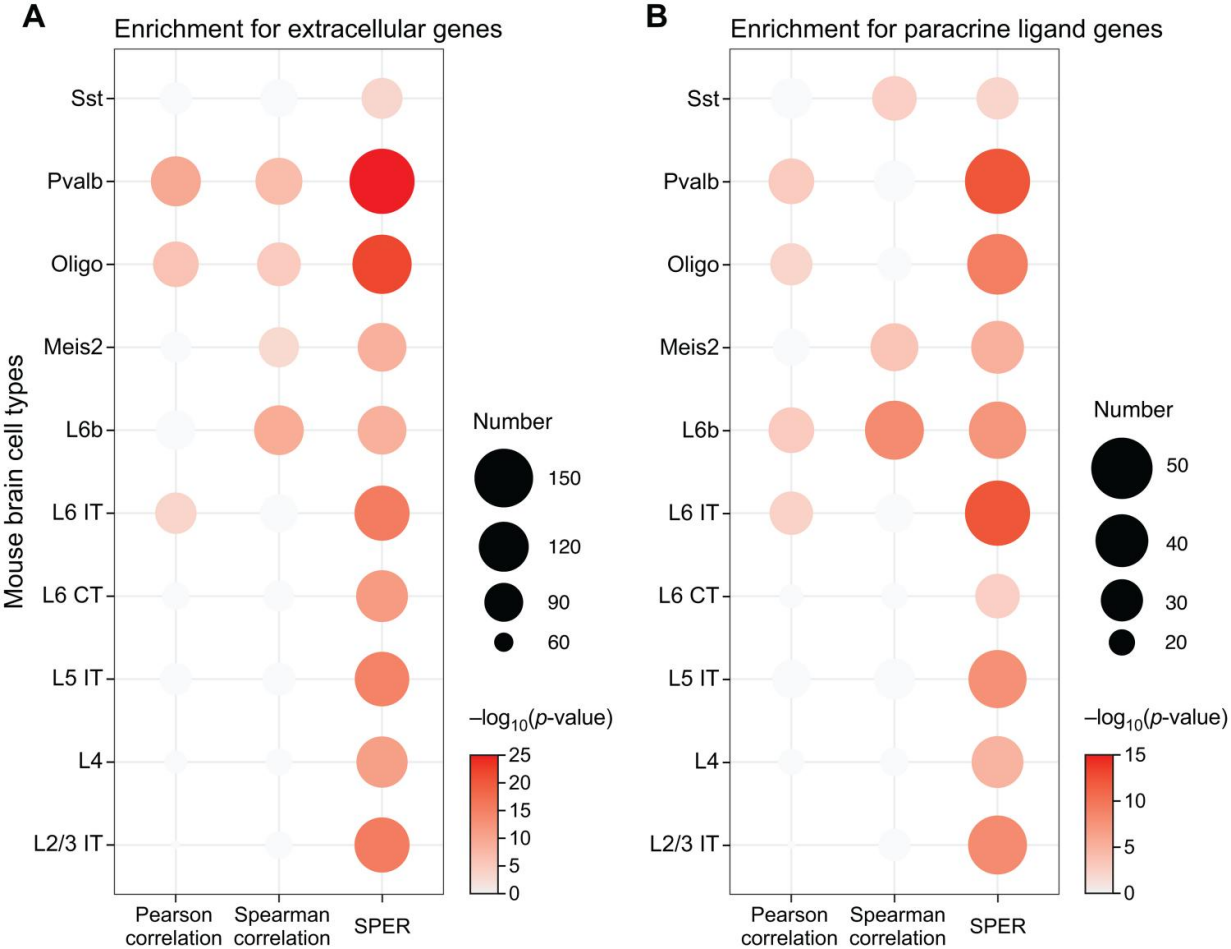
SPER’s top-ranked genes are enriched for both extracellular genes and known paracrine ligands. Dot plots visualize hypergeometric enrichment analysis of extracellular (A) and known paracrine ligand signals (B) within candidate gene sets. Extracellular and ligand genes annotations were collected from COMPARTMENTS, FANTOM5, and CellPhoneDB databases respectively (see Methods). The candidate sets include genes whose scores for each spatial dependency metric (x-axis) are above than the 95% percentile of all genes.

If genes spatially associated with cell type composition participate in paracrine interactions, they should encode proteins involved in intercellular communication. To test this, we assessed whether putative paracrine transcripts identified by SPER, and other methods were significantly enriched for (i) genes known to be secreted into the extracellular space and (ii) known paracrine ligands (Fig. 4). We set a threshold at the 95th percentile of SPER and other metric scores to identify significant transcripts. SPER-identified transcripts were significantly enriched (*P* < .001) for both extracellular genes and paracrine ligands (Fig. 4A and B) cross all 10 neuronal cell types in the mouse brain dataset. In contrast, Pearson and Spearman correlations showed significant enrichment (*P* < .05) for only three and four cell types (extracellular genes, Fig. 4A) and four and three cell types (paracrine ligands, Fig. 4B), respectively. With few exceptions, SPER identified more of both types of transcripts compared to Pearson and Spearman correlations. Lastly, we performed a sensitivity analysis to confirm the utility of co-kriging in denoising our compositional data (Section 2). We found that while transcripts highly ranked by SPER without co-kriging still showed enrichment, the addition of co-kriging improved the enrichment for both extracellular location and known paracrine ligand activity for the majority of cell types, demonstrating its utility in improving SPER’s sensitivity (Fig. 4, available as supplementary data at *Bioinformatics Advances* online). Together, these results demonstrate that candidate paracrine transcripts identified by SPER have three important characteristics: (i) they are predominantly expressed by a neighboring cell type, not the target cell, but they are nonetheless spatially related to the target cell’s composition, and they are also statistically enriched for (ii) genes known to be secreted into the extracellular space, and (iii) genes known to participate as ligands in paracrine interactions.

### 3.5 SPER detects single- and multi-source putative paracrine signals in mouse brain

**Figure 5:**
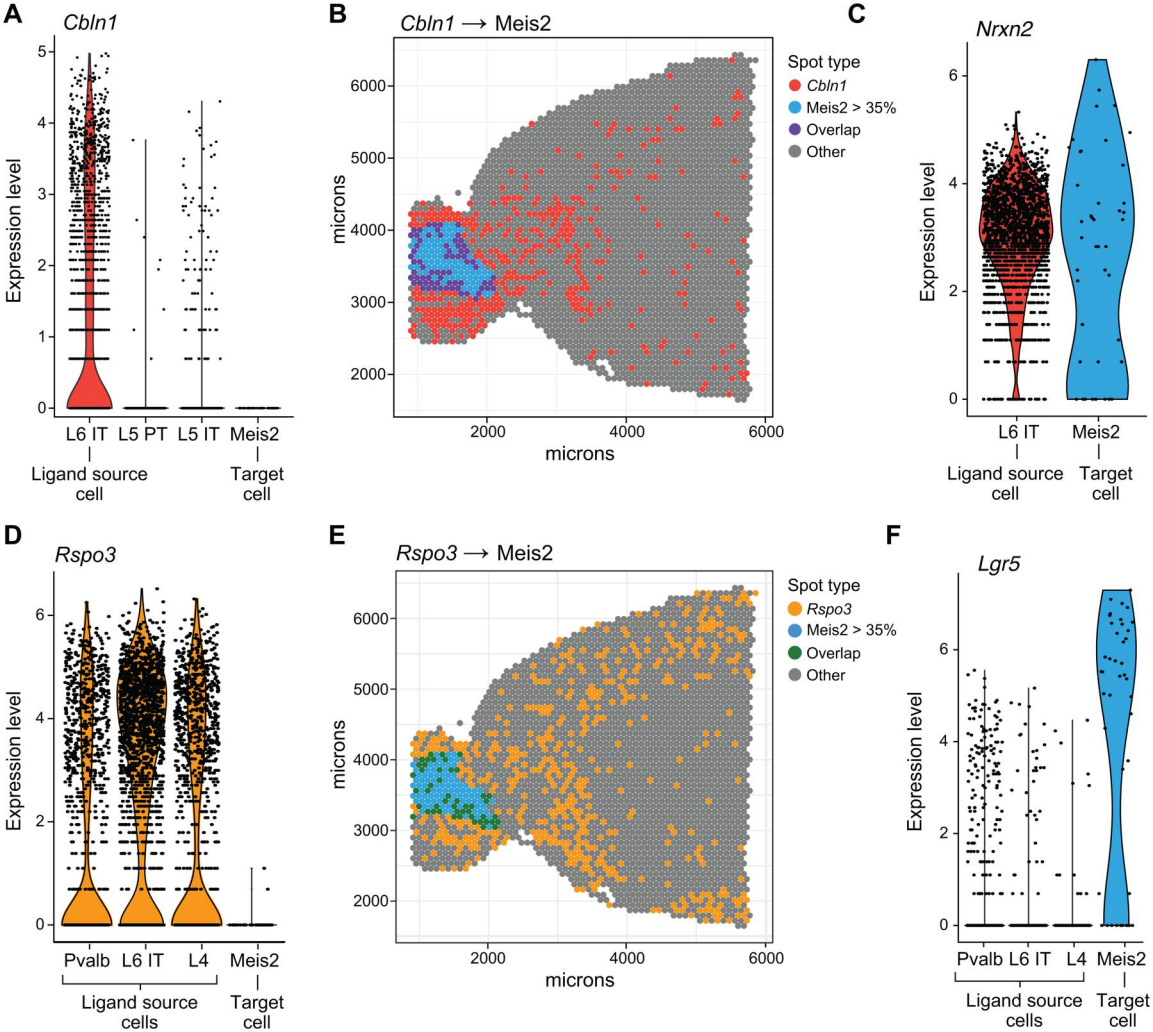
SPER detects single-source (Cbln1) and multi-source (Rspo3) paracrine signals in mouse brain. (A, D) Violin plots show expression levels (*y*-axis) of Cbln1 and Rspo3 in relevant mouse brain cell types (*x*-axis) from reference scRNA-seq data. L6 IT, L5 PT, L5 IT, L4, and Pvalb neurons are the neighboring cell types of Meis2+ neurons. (B, E) ST plots visualize the spatial dependence between transcript-target cell type pairs Cbln1/Meis2+ neurons (B) and Rspo3/Meis2+ neurons (E). The color of spots (color legend) shows whether Cbln1 (B) or Rspo3 (E) is detected, a proportion of Meis2 cell larger than 35%, both (overlap), or otherwise. (C, F) The expression levels of the relevant cognate receptor Nrxn2 (C) and Lgr5 (F) in the reference scRNA-seq data.

SPER identified a set of putative ligand genes with strong spatial associations to target cell type compositions. Across all cell types, 5569 cell type–ligand pairs exceeded the 95th percentile of SPER scores. Among these, 250 exhibited substantial expression of the cognate receptor in the target cell types, resulting in 506 ligand-receptor pairs potentially involved in regulating brain tissue composition. For example, Cbln1 (precerebellin) and Meis2+ neurons [a cell type defined by expression of the homeobox transcription factor Meis2 (Tasic *et al.* 2016, Frazer *et al.* 2017)] showed a strong spatial association, but also non-overlapping spatial distribution, indicating possible paracrine compositional regulation (Fig. 5B). Notably, it was not identified by Pearson correlation, ranking 349th (*R* = 0.32). On the other hand, using SPER we found that the Cbln1-Meis2+ neurons pair had the 4th highest score among all Meis2+ neuron-related signals where the appropriate cognate receptor was also expressed. Consistently, the scRNA-seq data showed that Cbln1 is only lowly expressed in Meis2+ neurons but is indeed highly expressed by the neighboring L6 IT neurons (Fig. 5A). In this case, reference scRNA-seq data showed that the paracrine signal is expressed only by this single source cell type. We examined Nrxn2, a receptor for Cbln1 (Fig. 5C), and found as expected that it was expressed on Meis2+ neurons, supporting the possibility of Cbln1 acting on them. In cerebellum, Cbln1 is known to play an important role in the Purkinje cell synapse formation (Ito-Ishida *et al.* 2014) as well as the growth and guidance of axon in multiple neural regions within mouse embryos (Han *et al.* 2022), but to our knowledge, its role as a paracrine signal in the motor cortex has not yet been described.

Paracrine signals can of course also be derived from multiple cell types. For example, SPER identified Rspo3, which encodes the Wnt ligand R-Spondin 3. Rspo3 is produced by several source cells: Pvalb+, L6 IT, and L4 neurons (Fig. 5D), and shows a non-overlapping spatial association with the identified target cells, again Meis2+ neurons (Fig. 5E). While Rspo3 is identified as a significant putative signal by SPER, it cannot be found as a paracrine signal using any correlation metrics (Table 1, available as supplementary data at *Bioinformatics Advances* online). We tested for expression of the cognate receptor for Rspo3 in Meis2 cells and found that Lgr5 (Leucine-rich repeat-containing G-protein coupled receptor 5) was strongly and selectively expressed in the target cell type, consistent with an active paracrine interaction (Fig. 5F). This suggests that Rspo3, released by Pvalb, L6 IT and L4 cells, may serve as a paracrine regulator to Meis2 cells, activating the Wnt signaling pathways, which could potentially shape the spatial compositions of Meis2+ neurons. While this interaction is well-studied in the intestine (Barker *et al.* 2007), to our knowledge its role in the mouse motor cortex is not yet known. The link between Rspo3 and Meis2 cells, therefore, provides a representative example of multi-source paracrine compositional regulators that can be discovered using SPER.

### 3.6 SPER enables discovery of paracrine signals from established human lung and breast tumor spatial dataset

**Figure 6:**
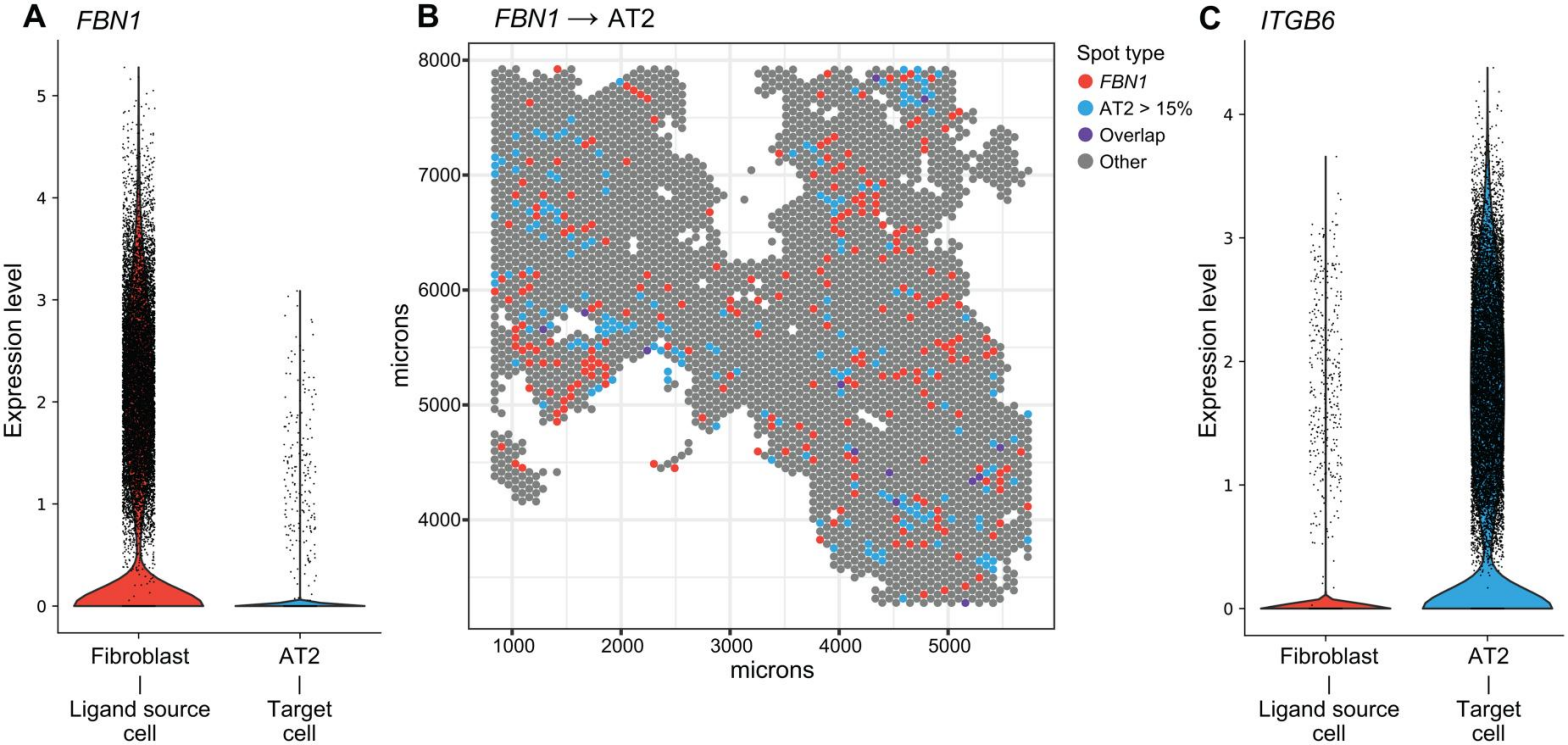
SPER detects non-overlapping paracrine signals (FBN1) in human lung. (A) Violin plots show expression levels (*y*-axis) of FBN1 in relevant human lung cell types (*x*-axis) from reference scRNA-seq data. (B) ST plots visualize the spatial dependence between transcript-target cell type pairs FBN1/AT2 cells. The color of spots (color legend) shows whether FBN1 is detected, a proportion of AT2 cell larger than 15%, both (overlap), or otherwise. (C) The expression levels of the relevant cognate receptor ITGB6 in the reference scRNA-seq data.

To validate SPER’s sensitivity to detect such paracrine signals, we also applied it to a second published ST dataset of the human lung (Madissoon *et al.* 2023). The processed Visium spatial transcriptomics data was deconvoluted by cell2location (Kleshchevnikov *et al.* 2022), based on the paired reference scRNA-seq data with a total of 1147 genes in approximately 200 thousand cells. The deconvolved ST data (Fig. 6) contains 3234 spots and the relative compositions of 80 annotated cell types. We applied SPER (Section 2) to identify potential paracrine regulators of human lung cell type abundances (Table 2, available as supplementary data at *Bioinformatics Advances* online). In addition to identifying many known paracrine interactions—such as club cell-derived SCGB3A2 and alveolar macrophage abundance, via the MARCO receptor (Blackburn *et al.* 2023) or basal epithelial cell-derived CXCL14 and B cell abundance, via the receptor CXCR4 (Rodriguez *et al.* 2018)—SPER identified a novel fibroblast-derived signal: fibrillin-1 (FBN1), an extracellular matrix (ECM) protein (Li *et al.* 2023), was spatially associated with the composition of alveolar type II (AT2) cells (Fig. 6A and B). The association for FBN1 and AT2 cells was very strong, ranking in the 98th percentile of SPER scores (20th of 1147 tested genes), but both Pearson and Spearman correlation scores failed to capture this signal with *R* = 0.056 and ρ = 0.061, outside the top 200 ranked transcripts for both metrics. Consistent with a plausible paracrine signaling role of fibroblast-derived FBN1, integrin αvβ6 (ITGB6), an epithelial-exclusive integrin that acts as a receptor for FBN1, is strongly expressed on AT2 cells (Fig. 6C). In lung organoid models, diverse ECM proteins including FBN1 are known to be critical for differentiation and proliferation of AT2 cells (Hoffman *et al.* 2023), but to our knowledge, the direct link between fibroblast derived FBN1 and ITGB6 on AT2 cells has not been previously identified as active *in vivo*. Mutations in FBN1 result in increased availability of TGFβ and are linked to the congenital disease Marfan’s syndrome, the effects of which are predominantly cardiovascular, skeletal, and ocular, but also induce a respiratory phenotype (Tun *et al.* 2021) including lung lesions and spontaneous pneumothorax (Li *et al.* 2023).

## 4 Discussion

Sequencing technologies and computational methods for spatial transcriptomics are both developing rapidly, promising an exciting new wave of biological discoveries informed by spatial insight. However, despite the importance of cell type compositional changes, which are frequently the first analytical question asked in spatial and single-cell studies, and the potential of ST data to map them, and the well-established role of paracrine signals in dictating tissue composition (Meizlish *et al.* 2021), to our knowledge there has been no method to detect putative compositional regulators; SPER provides the first such method.

SPER is broadly applicable beyond 10X Genomics Visium, extending to other spot-based technologies like Slide-seq (Rodriques *et al.* 2019) and XYZeq (Lee *et al.* 2021) with no workflow modifications, requiring only a genes-by-location matrix, spot coordinates, and deconvolved cell-type compositions. Because SPER was developed to model gene–composition dependencies at the mesoscale, its design aligns most naturally with spot-level spatial transcriptomics platforms. For single-cell spatial technologies, such as CosMx (NanoString), MERSCOPE (Vizgen), and Xenium In Situ (10X Genomics), SPER can still be applied but requires preprocessing to treat each cell as a distinct spot. While the workflow remains functional, the computational demand could be significantly higher than for spot-based data.

A key consideration when applying SPER to multi-cell–level ST data is that its accuracy ultimately depends on the quality of the underlying cell type deconvolution results. This dependency is not unique to SPER but is shared by all analytical frameworks built on spot-level ST data. In practice, we found that SPER is robust to moderate deconvolution noise, as its spatial modeling through co-kriging effectively integrates information from neighboring spots, thereby reducing the influence of local estimation errors (Tolosana-Delgado *et al.* 2019, Payares-Garcia *et al.* 2024).

It is important to note that SPER is conceptually distinct from existing ligand–receptor (LR) analysis frameworks (Shao *et al.* 2022, Tanevski *et al.* 2022, Li *et al.* 2023). LR tools rely on curated gene–gene interaction databases to infer communication between cell types, whereas SPER instead quantifies spatial dependencies between gene expression and cell-type abundance directly from the data, without requiring prior knowledge of receptor relationships. This design enables SPER to identify diffusible or niche-structuring factors—such as cytokines (Altan-Bonnet and Mukherjee 2019) or extracellular matrix components (Bowers *et al.* 2010)—that influence the recruitment or exclusion of specific cell types even in the absence of annotated ligand–receptor interactions. This capability is exemplified by SPER’s identification of fibroblast-derived FBN1 as a putative regulator of alveolar type II (AT2) cells in the human lung dataset, illustrating its ability to reveal de novo paracrine candidates beyond curated LR lists. While direct benchmarking against LR-based methods would therefore not be meaningful, these approaches are complementary. For instance, genes highlighted by SPER as candidate paracrine regulators could be further investigated through downstream LR analysis to pinpoint potential receptor-mediated pathways. Together, such analyses can provide a more comprehensive understanding of spatially organized cell–cell communication in tissues.

Algorithms for paracrine signal inference like SPER face two related challenges given the complexity of receptor ligand interactions. First, while SPER can identify significant receptor-ligand interactions, it relies on transcriptional data and thus only generates hypotheses rather than definitive physiological evidence, requiring experimental validation. As demonstrated in the mouse brain, SPER can uncover both known and novel interactions, making it a valuable first step in identifying intercellular circuits underlying tissue structure and transformation. Second, since many paracrine interactions remain unannotated, SPER does not exclude unclassified transcripts but ranks all transcript-cell type pairs, enabling the discovery of previously unknown regulatory signals, even when receptor identity is uncertain or undetected due to technical limitations.

Taken together, our results demonstrate that the application of SPER to ST data has the potential to identify which paracrine signals are involved in the intercellular circuits controlling homeostatic tissue composition, stress responses, tissue inflammation, and perhaps most importantly, pathological tissue transformations associated with disease. Identification of these signals is a critical step toward deepening systemic understanding of these complex processes and pinpointing novel biomarkers and potential therapeutic targets.

## Supplementary Material

vbag011_Supplementary_Data

## Data Availability

An R package to implement the SPER algorithm is available for download at github.com/TianxiaoNYU/SPER. Extracellular gene set in mouse is available from the COMPARTMENTS database: https://compartments.jensenlab.org/Downloads; scRNA-seq reference data is obtained from Allen Institute for Brain Science: https://portal.brain-map.org/atlases-and-data/rnaseq; Visium spatial transcriptomics data from mouse brain is available from 10X Genomics dataset: https://www.10xgenomics.com/resources/datasets/mouse-brain-serial-section-1-sagittal-anterior-1-standard-1-0-0. Human lung scRNA-seq and Visium data are available from Lung Cell Atlas: https://5locationslung.cellgeni.sanger.ac.uk/. Human breast cancer Visium dataset are available at: https://zenodo.org/records/4739739, and the scRNA-seq reference can be found at: https://singlecell.broadinstitute.org/single_cell/study/SCP1039. R markdown tutorials to help generate the main results of this article using SPER package, as well as all other necessary data files in this study can be found at: https://www.dropbox.com/scl/fo/pep07jjid71rr72voeeik/h?rlkey=2agyojo1ugf5l61bqtal6hjy0&dl=0.
