# Supplementary material for: Discovering paracrine regulators of cell type composition from spatial transcriptomics using SPER
Source: Bioinform Adv. 2026 Jan 19;6(1):vbag011. doi: 10.1093/bioadv/vbag011 (PMC12895071; doi:10.1093/bioadv/vbag011)
Supplement: vbag011_Supplementary_Data [file vbag011_supplementary_data.zip › Supplementary_Figures.docx]

**SUPPLEMENTARY FIGURES:**

**
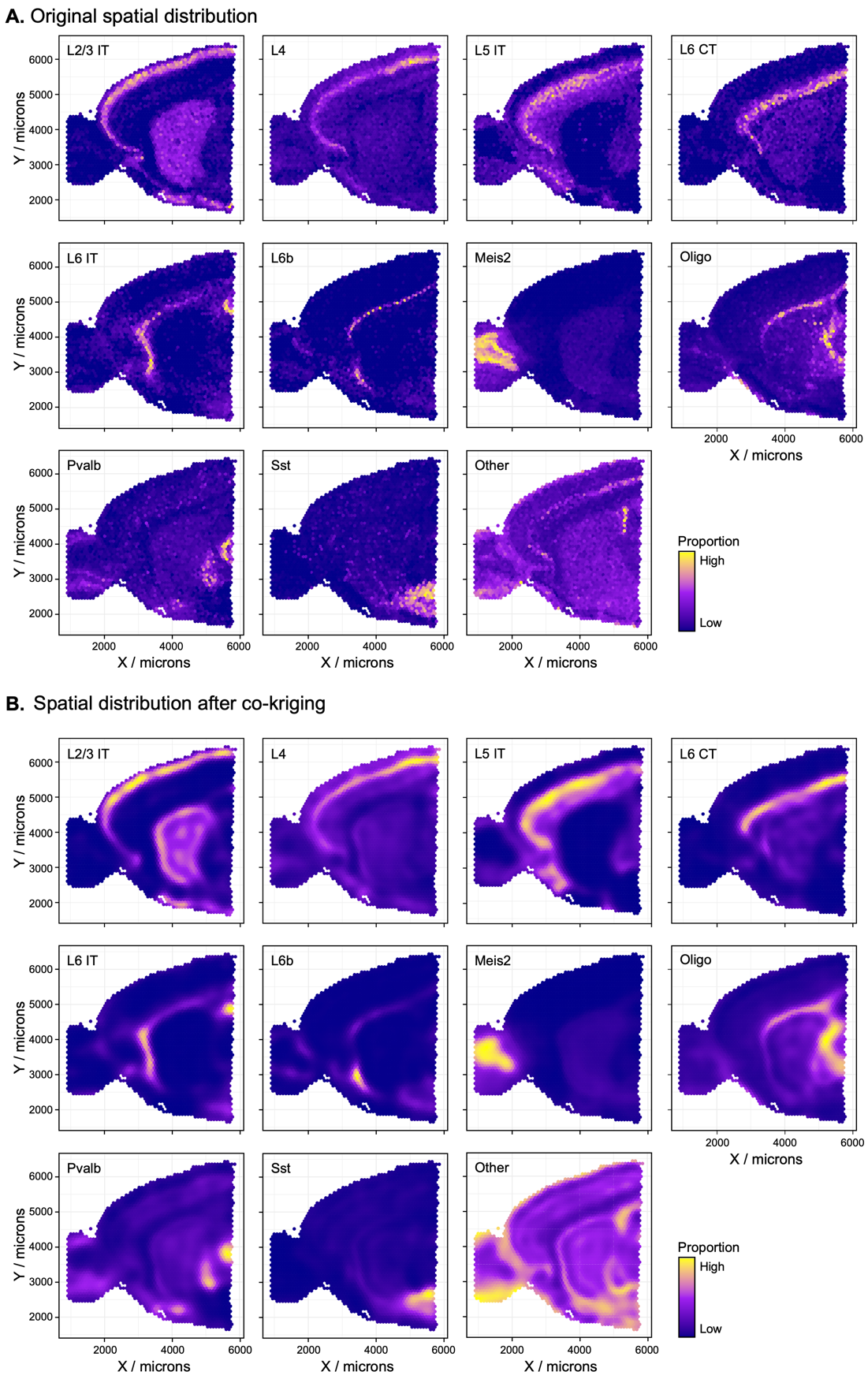
**

**Supplementary Figure 1. Co-kriging enables SPER to effectively denoise spatial compositional data**. Spatial distribution of composition of primary cell types decomposed by RCTD in full mode before (A) and after co-kriging modeling (B). The top ten most abundant cell types are modeled individually, and the other 11 cell types are summed as the ‘Other’ category. In each subplot, X and Y-axis represent the physical coordinates of spots, and the color represents the normalized proportion of the relevant cell type.

**
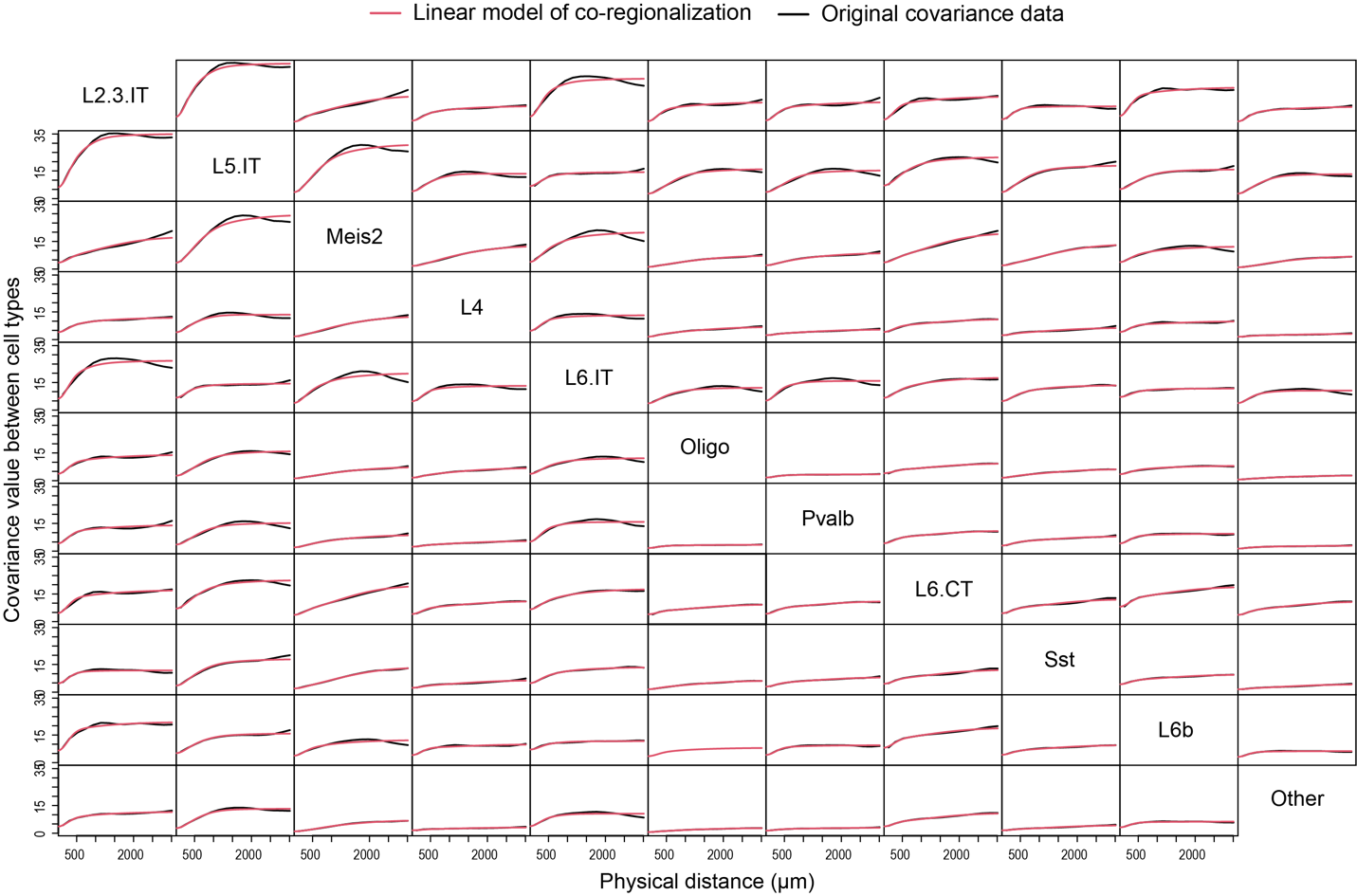
**

**Supplementary Figure 2. Co-kriging fits visualized as variograms**. Co-kriging method fits on the ten neuronal cell types from an example dataset from mouse brain. A compositional linear model of co-regionalization (red line) with a ‘nugget’ effect term for short scale randomness and three Gaussian variogram terms to model effects at different ranges (600, 1300, and 3000 μm) showed excellent fit to the original covariance data (black line) collected and calculated at 15 uniformly distributed lags. The labels at the diagonal identify the cell type for each row and column, while ‘Other’ represents the sum of all remaining cell types. The x-axis in each subpanel represents the physical distance in μm, and y-axis represents the relative value of the covariance between each pair of cell types.

**
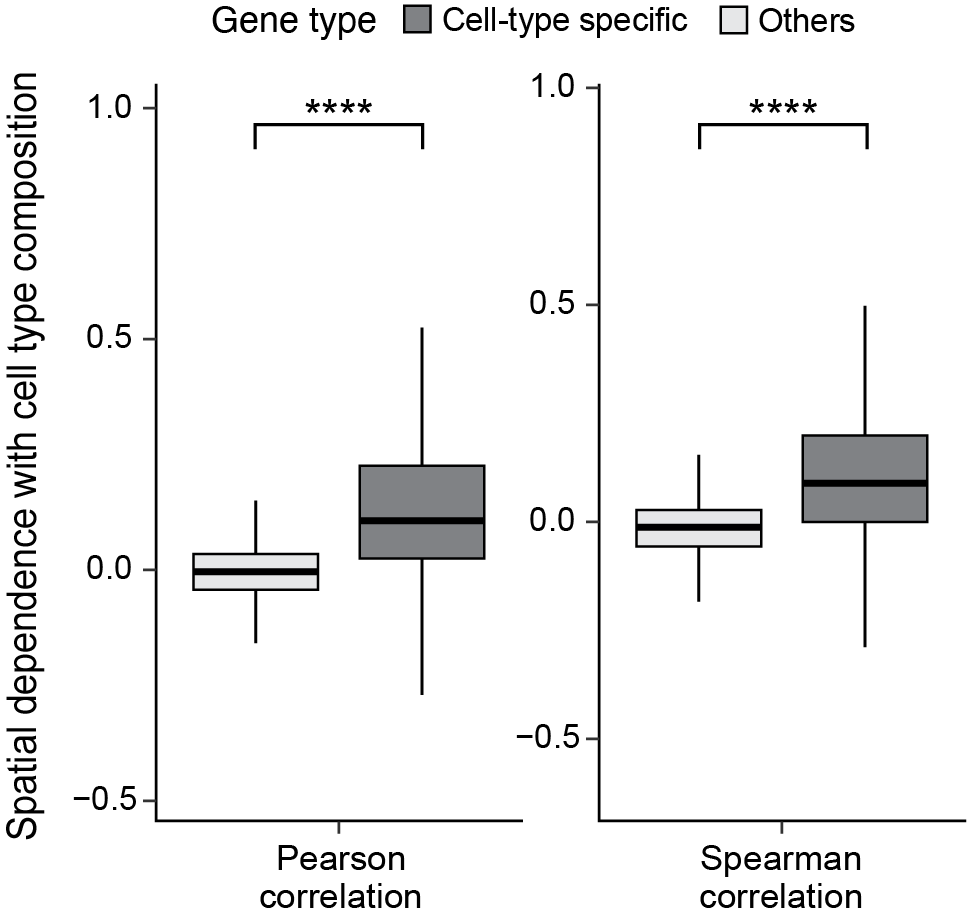
**

**Supplementary Figure 3. Pearson and Spearman correlations detect marker genes with overlapping spatial dependency.** Boxplots showing Pearson and Spearman correlations between cell type spatial compositions and gene’s spatial distribution. Marker genes for each cell types are selected from the scRNA-seq reference data by MAST. The four-star labels represent the difference (adjusted p-value < 10^-4^) in the measurement scores between the marker and non-marker groups.


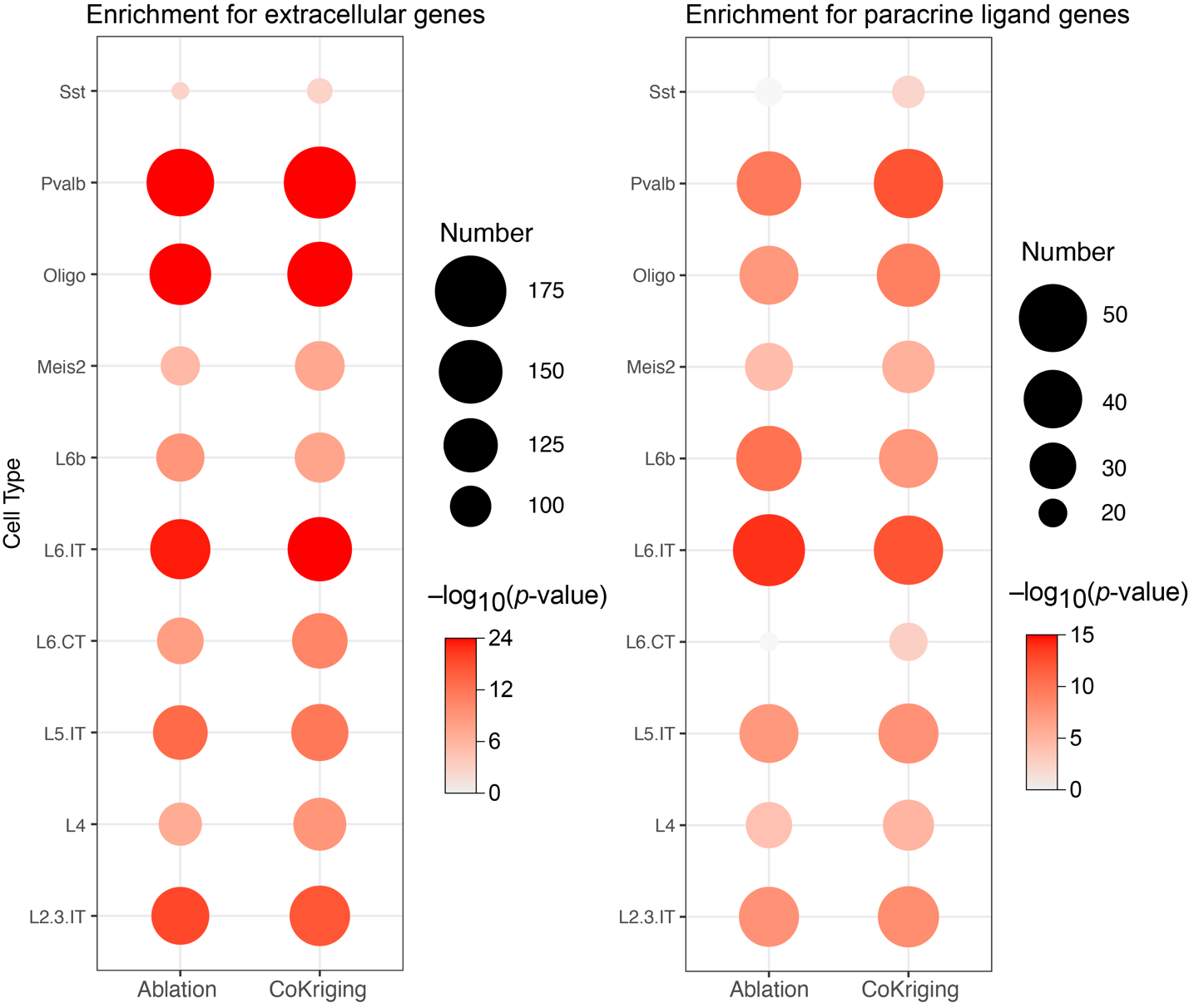


**Supplementary Figure 4. Ablation study demonstrates SPER detect more paracrine ligand genes with co-kriging.** Dot plots visualize hypergeometric enrichment analysis of known paracrine ligand signals with and without co-kriging analysis. Ligand genes annotations were collected from COMPARTMENTS, FANTOM5, and CellPhoneDB databases respectively (see **Methods**). The candidate sets include genes whose scores for each spatial dependency metric (x-axis) are above than the 95% percentile of all genes. The size of dots represents the number of extracellular or ligand genes in the given gene set. The color represents the significance the enrichment in the set (hypergeometric test), non-significant (*p*>0.05) overlaps are shown as light grey. With co-kriging, the number of transcripts in both categories increased for most cell types, with 9 out of 10 showing an increase for extracellular location and 8 out of 10 for paracrine ligands.


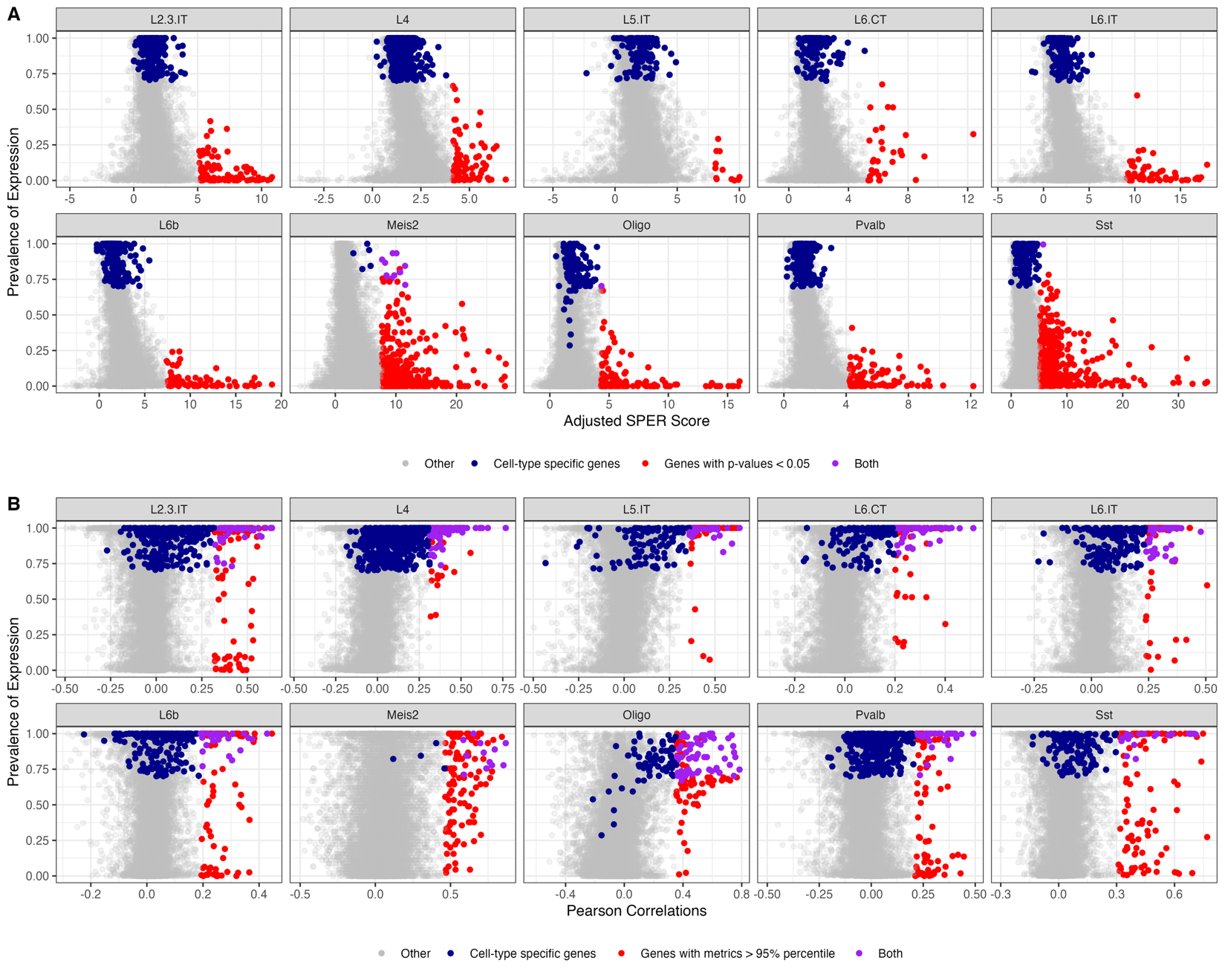


**Supplementary Figure 5. Comparison of trivial spatial associations and genuine putative regulators between SPER and Pearson correlation.** Scatter plots show the distribution of SPER (A) and Pearson correlation (B) scores (x axis) with the proportion of cells of each type (plots) in which each gene (points) is detected (y axis) in scRNA-seq data. The dark blue dots mark the marker genes for each cell type (found by MAST, details in Methods). Red dots stand for the genes whose p-values smaller than 0.05 (SPER) or correlations larger than 95% percentile (Pearson). Purple dots represent genes that meets both conditions.


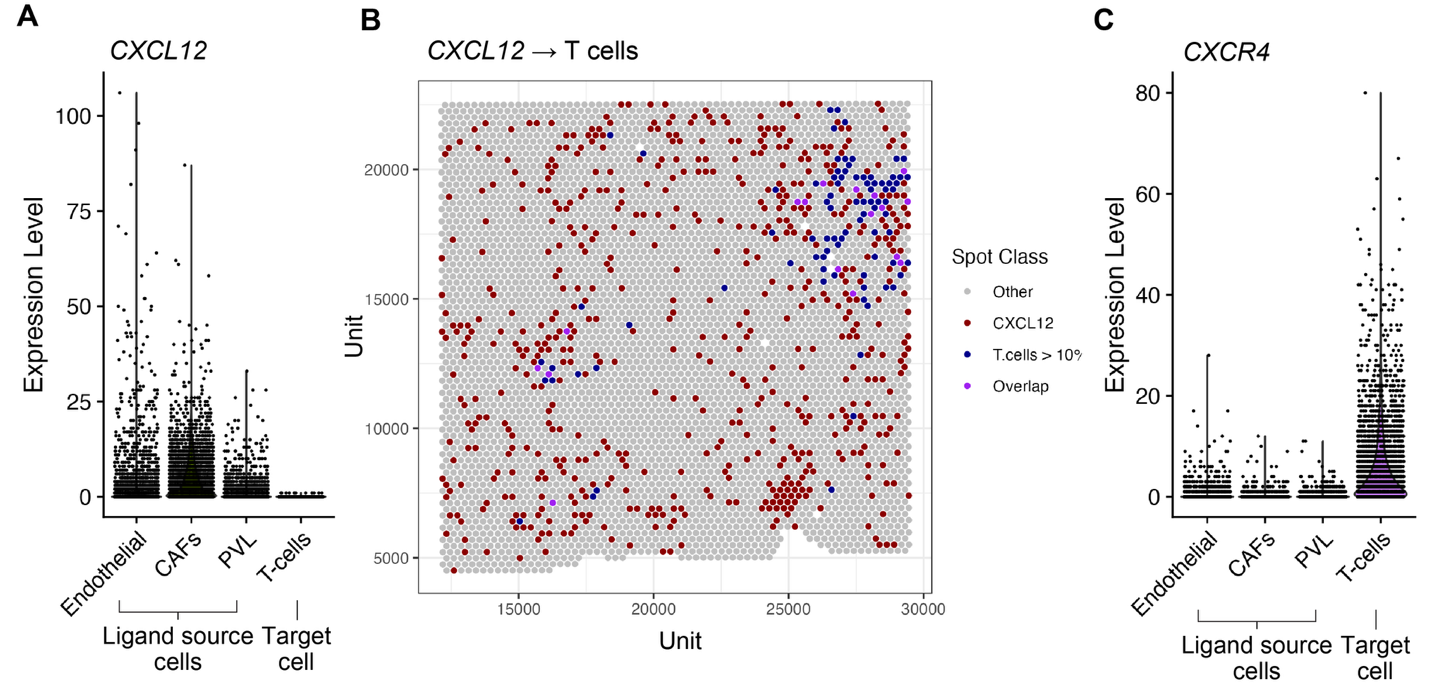


**Supplementary Figure 6. SPER detects non-overlapping paracrine signals (*CXCL12*) in human breast tumor dataset.** (**A**) Violin plots show expression levels (y-axis) of *CXCL12* in relevant human breast cell types (x-axis) from reference scRNA-seq data. (**B**) ST plots visualize the spatial dependence between transcript-target cell type pairs *CXCL12*/T cells. The color of spots (color legend) shows whether *CXCL12* is detected, a proportion of AT2 cell larger than 10%, both (overlap), or otherwise. (**C**) The expression levels of the relevant cognate receptor *CXCR4* in the reference scRNA-seq data.
